# Can transcriptome size and off-target effects explain the contrasting evolution of mitochondrial vs nuclear RNA editing?

**DOI:** 10.1093/jeb/voaf042

**Published:** 2025-05-05

**Authors:** Daniel B Sloan

**Affiliations:** Department of Biology, Colorado State University, Fort Collins, CO, United States

**Keywords:** constructive neutral evolution, mitochondrial, RNA editing, target specificity, transcriptome

## Abstract

Mitochondrial RNA editing has evolved independently in numerous eukaryotic lineages, where it generally restores conserved sequences and functional reading frames in mRNA transcripts derived from altered or disrupted mitochondrial protein-coding genes. In contrast to this “restorative” RNA editing in mitochondria, most editing of nuclear mRNAs introduces novel sequence variants and diversifies the proteome. This Perspective addresses the hypothesis that these completely opposite effects of mitochondrial vs. nuclear RNA editing arise from the enormous difference in gene number between the respective genomes. Because mitochondria produce a much smaller transcriptome, they likely create less opportunity for off-target editing, which has been supported by recent experimental work expressing mitochondrial RNA editing machinery in foreign contexts. In addition, there is recent evidence that the size and complexity of RNA targets may slow the kinetics and reduce efficiency of on-target RNA editing. These findings suggest that efficient targeting and a low risk of off-target editing have facilitated the repeated emergence of disrupted mitochondrial genes and associated restorative RNA editing systems via (potentially non-adaptive) evolutionary pathways that are not feasible in larger nuclear transcriptomes due to lack of precision.

## Introduction

**Figure 1.**
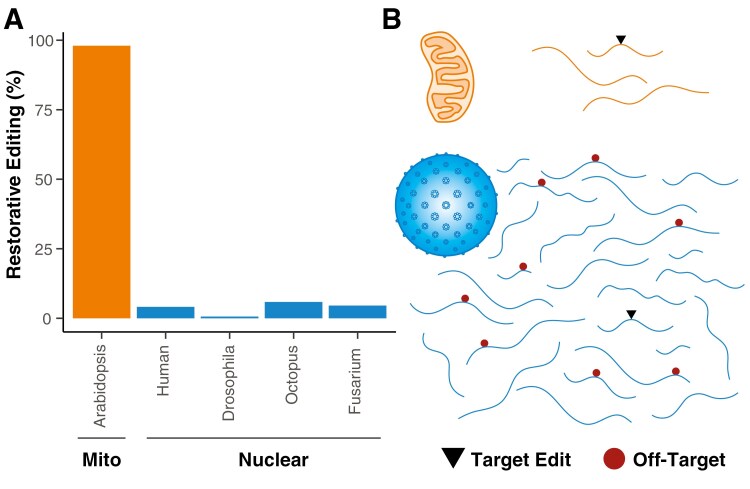
Contrasts between mitochondrial and nuclear RNA editing systems. (A) RNA editing events in mitochondria tend to restore ancestral protein sequence, whereas nuclear RNA editing tends to be diversifying and introduce derived variants. Data from Sloan (2017). Note that although there have been some reports of nuclear RNA editing in plants (Ruchika et al., 2021), they have not been well characterized with respect to restorative effects, and direct comparisons between nuclear and mitochondrial editing systems in the same organism have not been performed. (B) The larger number of nuclear genes and correspondingly larger nuclear transcriptome sizes (bottom) may dramatically increase the amount of off-target editing relative to the precise restorative systems found in mitochondria (top). Lines represent mRNA transcript with target and off-target edits indicated by black triangles and red circles, respectively.

RNA editing is an intriguing feature of gene expression in which the coding sequence of an mRNA transcript is modified prior to translation, resulting in a protein product that is inconsistent with the corresponding DNA sequence. This phenomenon has been documented across many independent evolutionary lineages with a diversity of molecular mechanisms that act via either base substitutions or insertions/deletions (indels) in RNA sequence (Knoop, 2011). The evolutionary forces responsible for the repeated origins of RNA editing are mysterious, and the potential roles of both adaptive and non-adaptive processes have been discussed extensively (Covello & Gray, 1993; Gommans et al., 2009; Zhang & Xu, 2022; Zhang et al., 2023). RNA editing patterns in mitochondrial vs nuclear genomes exhibit a striking contrast that further adds to this puzzle. Specifically, editing of mitochondrial transcripts largely restores protein sequences to the ancestral state and increases similarity to homologous proteins in related species, whereas editing of nuclear transcripts generally has a diversifying effect on protein sequences by introducing derived variants (Sloan, 2017). For example, a previous analysis found that 98% of RNA edits were restorative with respect to protein sequence in the mitochondria of the model angiosperm *Arabidopsis thaliana*, whereas >94% were diversifying in nuclear RNA editing systems from multiple animals and the ascomycete fungus *Fusarium graminearum* (Figure 1A). In many other organisms with mitochondrial RNA editing (see below), the effects of editing are even more restorative in the sense that they involve small indels that must be the correct length and location to even have an intact open reading frame. This Perspective explores the hypothesis that these opposite outcomes of mitochondrial vs nuclear RNA editing are due to the radical difference in size (i.e., sequence complexity) between mitochondrial and nuclear transcriptomes. Because mitochondrial genomes retain only dozens of genes (at most), their transcriptomes likely have a much lower propensity for “off-target” edits than their nuclear counterparts derived from thousands of genes (Figure 1B). As outlined below, this difference may have profound implications for the evolution of RNA editing.

For reasons that have never been entirely clear, mitochondria appear to be especially prone to evolve RNA editing. These editing systems often act on a large proportion of sites in mitochondrial transcriptomes, in some cases restoring functional reading frames to protein-coding genes that are essentially unrecognizable (“cryptogenes”) based on genomic sequence alone. Taxa with pervasive mitochondrial RNA editing include land plants (Takenaka et al., 2013), heteroloboseids (Yang et al., 2017), trypanosomes (Read et al., 2016), diplonemids (Kaur et al., 2020), dinoflagellates (Waller & Jackson, 2009), myxomycetes (Horton & Landweber, 2000), and calcareous sponges (Lavrov et al., 2016). The RNA editing mechanisms in land plant mitochondria are also found in their plastids (Takenaka et al., 2013). The restorative effects of RNA editing in these organelles necessitate a high degree of target specificity, but the mechanisms that achieve this specificity differ greatly across lineages. For example, land plant editing sites are determined by an enormous family of nuclear-encoded pentatricopeptide repeat (PPR) proteins that target specific RNA sequences based on a PPR “binding code” in mitochondria and plastids (Barkan et al., 2012; Fujii & Small, 2011; Gerke et al., 2020), whereas trypanosome mitochondrial genomes contain large numbers of “minicircles” encoding guide RNAs that are responsible for editing specificity (Aphasizhev & Aphasizheva, 2014; Read et al., 2016).

The most widely studied examples of nuclear RNA editing include cytosine-to-uracil (C-to-U) and adenosine-to-inosine (A-to-I) base substitutions, both of which are mediated by deaminase activity. In animal systems, C-to-U and A-to-I editing are performed by APOBEC and ADAR protein families, respectively (Nishikura, 2010; Pecori et al., 2022). Some fungi also exhibit A-to-I editing of nuclear transcripts, but this activity has evolved independently and is mediated by different enzymatic machinery (Feng et al., 2024). Because the vast majority of nuclear mRNA edits are diversifying rather than restorative (Sloan, 2017), their recoding activity is thought to be an evolutionary adaptation that produces alternative protein sequences that can be regulated in a tissue, development, or environment-specific fashion (Gommans et al., 2009; Zhang et al., 2023). In some cases, the importance of specific editing targets has been identified, such as the APOBEC1-mediated introduction of a stop codon in apolipoprotein B (ApoB) that results in two isoforms differing in length and relative abundance across human tissues (Blanc & Davidson, 2010). However, the number of identified editing sites has grown tremendously, and comparative analyses suggest that a substantial proportion of this editing is simply the result of off-target “misfiring” of editing machinery (Liu & Zhang, 2018; Xu & Zhang, 2014).

The problem of off-target editing poses a potential explanation for why so many eukaryotic lineages have evolved a dependence on extensive restorative RNA editing in mitochondria but not in the nuclear transcriptome (Figure 1B). For example, if the probability of promiscuous activity on a random off-target sequence were held constant, a species such as *Arabidopsis thaliana* would have ~1000-fold fewer off-target edits in the mitochondrial transcriptome than the nuclear transcriptome given the difference in total protein-coding gene sequence length between the genomes. Therefore, it is possible for highly precise editing systems to evolve in mitochondria, as suggested by the overwhelming majority of mitochondrial edits being restorative (Figure 1A). In contrast, even if restorative editing is an important function at some specific sites in nuclear editing systems, any signal from this function is likely to get swamped by off-target editing that leads to largely random diversification of protein sequences. Of course, mitochondrial systems are not entirely immune to off-target editing. For example, the small number of synonymous sites in plant mitochondrial genomes that are subject to RNA editing show signatures of being off-target and largely neutral misfirings of editing machinery (Mower & Palmer, 2006; Sloan et al., 2010) and use of artificial “designer” PPRs has produced detectable off-target mitochondrial editing in some cases (Manavski et al., 2025). However, because of the apparent rarity of these off-target effects, they likely make little contribution to the overall pattern of editing in mitochondria.

The ability to transfer editing machinery into other organisms or cellular compartments is providing exciting opportunities to directly compare the extent of off-target effects. For example, recent studies have taken a pair of mitochondrial RNA editing factor (PPR56 and PPR65) from the moss *Physcomitrium patens* and retargeted them to the moss cytosol or heterologously expressed them in *E. coli* or human cells (Lesch et al., 2022; Oldenkott et al., 2019; Thielen et al., 2024). In moss mitochondria, these two PPR proteins perform precise C-to-U RNA editing at a total of just three sites. They were also effective at editing these same sites when their native targets were co-expressed in the foreign systems. However, expression of these two PPRs yielded extensive off-target editing (~100 sites in *E. coli* mRNA transcripts and ~1,000 sites in both moss and human nuclear mRNA transcripts). These experiments offer an elegant illustration of how the specificity of RNA editing within mitochondria can be lost in the context of much larger bacterial or nuclear transcriptomes.

In addition to the risk of off-target editing, the size and complexity of transcriptomes may affect the kinetics and efficiency of on-target editing. For example, recent in vitro assays have shown that the time required for RNA binding to a cognate sequence by a PPR increases with the length and secondary structure of the RNA transcript (Marzano et al., 2024). Therefore, achieving complete RNA editing in a large and complex transcriptome may require high levels of expression of editing factors. In turn, overexpression of these factors may exacerbate the risk of off-target events, creating a natural trade-off between on-target efficiency and rates of off-targeting editing that could also explain the rarity of restorative editing outside of organelles (Marzano et al., 2024).

Although the limited risk of off-target effects is a potential explanation for why restorative RNA editing systems *can* exist in mitochondria, it does not explain why mitochondrial RNA editing systems *do* evolve so often. Indeed, the *raison d’être* of RNA editing is a longstanding curiosity in the field of molecular evolution. Because restorative editing essentially has the effect of reversing DNA mutations at the RNA level, it might appear to be an adaptive “mutational buffer” (Borner et al., 1997). Indeed, this explanation could provide an alternative hypothesis for why RNA editing is so common in mitochondria because mitochondrial mutation rates are very high in many eukaryotic lineages, but it presents both conceptual and empirical difficulties. First, the evolution of site-specific editing as a *response* to deleterious mutations would require mutations that are sufficiently harmful and at high enough frequency in the population to create a strong selection pressure for restorative editing. This requirement presents a potential Catch-22 because a strongly deleterious mutation is unlikely to overcome selection and spread to high frequency. Second, RNA editing is prevalent even in lineages with low mitochondrial mutation rates, such as land plants (Wolfe et al., 1987). In fact, high mitochondrial mutation rates are associated with the loss/lack of editing in some cases (Lynch et al., 2006; Parkinson et al., 2005; Sloan et al., 2010). Adaptive effects of proteome diversification and gene regulation are another commonly invoked explanation for the evolution of RNA editing (Gommans et al., 2009; Zhang et al., 2023). However, there is little evidence to date for these roles in mitochondrial systems where a given edit is often observed in all or nearly all transcript copies and partial editing has not been tied to key regulatory roles (Rüdinger et al., 2009).

An alternative non-adaptive model was posed for the origins of mitochondrial RNA editing soon after its discovery, and this model has since been generalized to the concept of constructive neutral evolution (CNE) (Covello & Gray, 1993; Muñoz-Gómez et al., 2021; Stoltzfus, 1999). Under a CNE hypothesis, the (potential for) site-specific editing activity predates the deleterious mutation and, because it is already in place, this activity makes an otherwise deleterious mutation effectively neutral and able to spread by genetic drift. If the mutated allele rises to a high frequency in the population, the site-specific editing activity would then become essential and maintained by selection. This hypothetical process is considered neutral or non-adaptive because the increase in molecular complexity occurs without ever boosting fitness or reversing a fitness decline in the population. Importantly, the CNE model does not suffer from the aforementioned challenges that undermine hypotheses based on mutational buffering.

The possibility that the origin and proliferation of RNA editing in the mitochondria of many eukaryotic lineages may have progressed via CNE could also address a question that naturally arises from the central hypothesis of this Perspective. If the barrier to restorative editing systems in nuclear transcriptomes is related to the inaccuracy of RNA editing and the risks of off-target effects, why couldn’t nuclear RNA editing systems simply evolve higher fidelity? After all, other forms of post-transcriptional modifications such as intron splicing are widespread in nuclear transcriptomes with seemingly sufficient accuracy. Assuming that it is biochemically possible to achieve the necessary combination of efficiency and accuracy for RNA editing, it would presumably involve refinement of editing activity that initially had lower fidelity. In the context of large and complex transcriptomes, the harm associated with inaccurate editing activity may be too great for the activity to persist long enough for it to facilitate the neutral spread of otherwise deleterious mutations that eventually lock in the need for editing.

In summary, the combination of CNE and low risks of off-target effects may make mitochondria a hotspot for the evolution of restorative RNA editing. In contrast, selection for regulated production of alternative protein isoforms in combination with extensive non-adaptive off-target effects appears to better explain the patterns of nuclear RNA editing (Liscovitch-Brauer et al., 2017; Xu & Zhang, 2015; Zhang et al., 2023). Therefore, the strikingly opposite effects of RNA editing that distinguish mitochondrial and nuclear systems may ultimately reflect something as simple as their large differences in genome size and gene content.

## Data Availability

No new data were generated in writing this Perspective.
